# Chloroform Applied Locally in Fractures

**Published:** 1852-05

**Authors:** H. N. Hurlbut

**Affiliations:** Chicago


ART. V.—*Chloroform applied locally in Fractures and Dislocations.*

*Messrs. Editors*—PERMIT me to call the attention of the profession to the local application of chloroform to remove pain and induce insensibility of parts to be operated upon. I have made use of it for two years past as a local remedy with the most happy results. In cases of comminuted fractures, producing as it does insensibility and relaxation of parts, it enables the surgeon to overcome the resistance of muscles, and to reduce the fractures without pain or suffering to the patient. In dislocations, I have no doubt its local application would be attended with the same happy results. I have used it in a similar manner in felons, neuralgia, &c. The best mode of application, I think, is to saturate a cloth with it, apply immediately, and over that a piece of oil silk (to prevent evaporation), allowing it to remain some four or five minutes, which will be sufficient. A second application may be necessary in fractures of the superior third of the femor, or in dislocation of the same bone.

The frequent deaths reported from the inhalation of chloroform has induced me to call attention to its local use. If in the hands of others it shall prove as safe and efficient a remedy as it has in mine, I shall be amply rewarded. H. N. HURLBUT, M.D.

CHICAGO, April 5, 1852.

